# Optimizing Injection Molding Tool Design with Additive Manufacturing: A Focus on Thermal Performance and Process Efficiency

**DOI:** 10.3390/ma18030571

**Published:** 2025-01-27

**Authors:** Deviprasad Chalicheemalapalli Jayasankar, Thomas Tröster, Thorsten Marten

**Affiliations:** Institute for Lightweight Design with Hybrid Systems (ILH), Automotive Lightweight Design (LiA), Paderborn University, Warburger Str. 100, 33098 Paderborn, Germany; thomas.troester@uni-paderborn.de (T.T.); thorsten.marten@uni-paderborn.de (T.M.)

**Keywords:** additive manufacturing (AM), injection molding, vacuum-assisted resin transfer molding (VA-LRTM), cooling channels, carbon fiber reinforced plastic (CFRP)

## Abstract

Injection molding plays a pivotal role in modern manufacturing, enabling the mass production of complex components with high precision. However, traditional tooling methods often face challenges related to thermal management, design constraints, and material efficiency. This study examines the use of additive manufacturing (AM) in the development and optimization of injection molding tools to overcome these limitations. A novel prototype was fabricated using AM techniques, incorporating integrated cooling channels and optimized lattice structures to enhance thermal performance and simplify the manufacturing process. Experimental validation demonstrated the prototype’s effective integration into a vacuum-assisted resin transfer molding (VA-LRTM) system without requiring modifications to existing tooling setups. The results showed significant improvements in temperature regulation, reduced cycle times, and consistent mechanical properties of the molded components compared to conventional approaches. By reducing the number of tool components and eliminating the need for support structures during manufacturing, AM also minimized material waste and post-processing requirements. This research highlights the transformative potential of additive manufacturing in injection molding tool design, offering increased flexibility, cost efficiency, and enhanced functionality to meet the evolving demands of modern industrial applications.

## 1. Introduction

**Figure 1:**
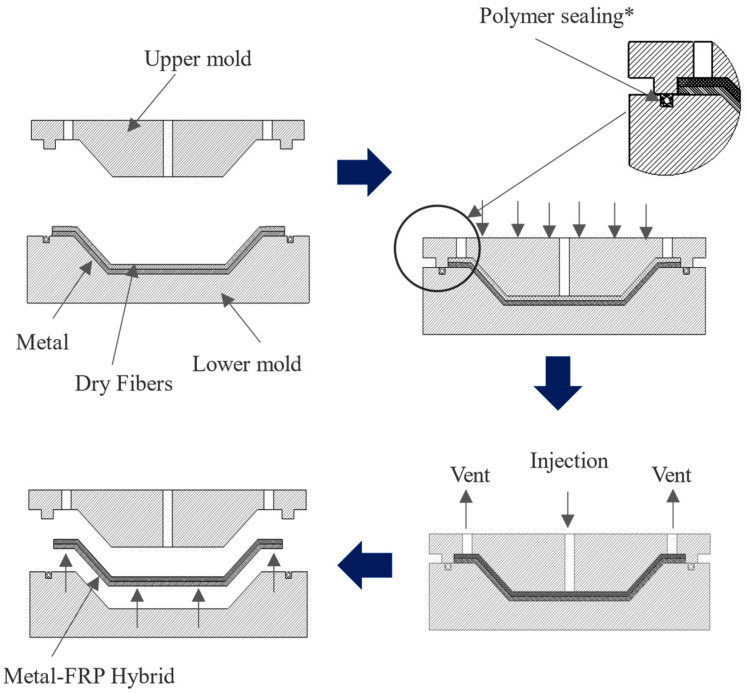
Block diagram showing the process cycle of Resin Transfer Molding (RTM). * Note: sealings are used only in vacuum RTM process.

Injection molding is a highly versatile and widely utilized manufacturing process that enables the production of complex, high-precision parts in large volumes [1,2,3]. It involves injecting molten material, typically polymers, into a mold cavity, where it cools and solidifies into the desired shape. Variants of this process, such as over-molding, micro-injection molding, and insert molding, address specific industrial requirements by combining multiple materials or achieving ultra-precise features [4,5,6]. In composite manufacturing, processes like resin transfer molding (RTM) extend injection molding’s principles to create fiber-reinforced polymer components, which offer exceptional strength-to-weight ratios [7,8,9,10]. In RTM, a liquid resin is injected into a closed mold containing a pre-placed fiber preform, where it impregnates the fibers and cures into a solid composite structure, as illustrated in Figure 1. The main difference between the injection molding and RTM processes lies in the requirement for sealing. The RTM process uses a matrix with significantly lower viscosity, typically ranging from 0.1 to 2 Pa·s, compared to the higher viscosity of polymer melts used in injection molding, which are often above 100 Pa·s [11,12,13,14]. This low viscosity makes the matrix prone to flowing, necessitating robust sealing to prevent resin leakage, contamination of the surrounding environment, loss of pressure, and potential tool damage during the molding process. Vacuum-assisted resin transfer molding (VA-RTM) further enhances RTM by using vacuum pressure to improve resin flow and fiber impregnation, ensuring high-quality, defect-free composite parts [15,16,17,18]. This advancement makes VA-RTM a favored technique in industries like aerospace, automotive, and wind energy, where lightweight and structural integrity are critical.

Nevertheless, achieving effective thermal management remains a key challenge in injection molding and its variants. Consistent thermal control is essential to ensure product quality, minimize cycle times, and maintain the structural integrity of molded components [19,20,21,22,23]. To regulate heat during the molding process, insulation materials such as glass fiber mats, ceramic layers, and other thermal barriers are commonly employed [24,25,26]. These materials are used to isolate heat in localized areas or reduce heat transfer between the mold and its surroundings, making them a widely adopted solution in conventional tooling systems. However, insulation materials come with inherent limitations. Their effectiveness relies heavily on precise placement and material properties, both of which can degrade under the thermal, mechanical, and chemical stresses encountered during repeated molding cycles. Many insulation materials are porous, making them susceptible to resin infiltration during molding processes like RTM, which compromises their insulating properties and durability. Additionally, they are prone to wear and require periodic replacement, increasing maintenance costs and causing downtime. The integration of these materials into the tool also adds complexity to the manufacturing process, as they must be carefully installed, further restricting design flexibility.

**Figure 2:**
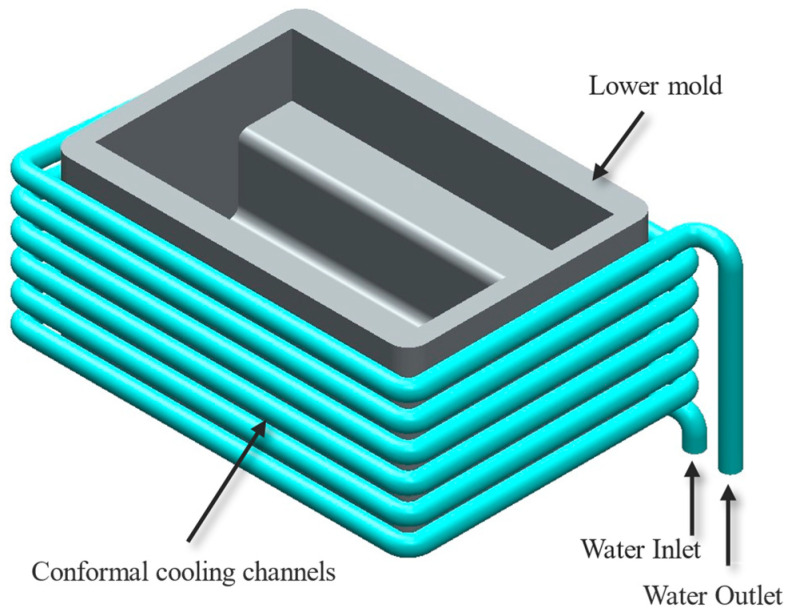
CAD showing a design of conformal cooling channel used in injection molding produced via conventional methods.

Cooling channels, on the other hand, have emerged as an alternative method for thermal management, offering superior regulation of heat flow and improved thermal consistency compared to insulation materials [27,28]. Traditional cooling channels are machined into molds and are effective for simple geometries [29,30]. However, they are inherently limited when it comes to complex mold designs. These channels are typically straight or linear, constrained by the machining processes used to create them, as shown in Figure 2. This inability to conform to intricate mold surfaces often results in uneven cooling, with hotspots forming in areas where heat dissipation is inadequate [31,32]. These thermal inconsistencies can lead to defects in the molded parts, such as warping, residual stresses, or dimensional inaccuracies, while also prolonging cycle times and increasing energy consumption. To address these issues, alternative manufacturing techniques were explored to evaluate their potential for providing optimized solutions.

Additive manufacturing (AM) provides an innovative solution to these limitations by enabling the creation of tooling with integrated cooling channels that eliminate the need for external insulation materials [33,34]. Unlike traditional methods, AM builds components layer by layer, allowing for design freedom and enabling the production of highly complex geometries that would be expensive to achieve otherwise. One of the most significant advantages of AM is the ability to fabricate conformal cooling channels that closely follow the mold’s geometry, ensuring uniform heat dissipation and eliminating the hotspots that traditional cooling methods struggle to address [35,36,37]. This not only improves part quality by reducing warping and residual stresses but also shortens cycle times, leading to increased production efficiency. Furthermore, AM allows for the integration of lattice structures, which function as internal thermal insulators, enhancing heat flow control while reducing material usage and tool weight [38,39]. These lattice structures provide controlled thermal barriers, offering a durable and maintenance-free alternative to conventional insulation while reducing weight and material usage. Additionally, AM allows for rapid prototyping and iterative design, which are particularly valuable in optimizing thermal performance during the tool development phase [40,41]. Tools can be customized to meet specific manufacturing requirements, such as integrating multiple functionalities like slots for connecting heating cartridges and thermocouples for localized temperature control and monitoring. The layer-by-layer fabrication approach also minimizes material waste, making AM a more sustainable option compared to traditional machining. In recent years, advancements in metal AM technologies have further expanded their application potential in industrial tooling. Techniques such as Directed Energy Deposition (DED), Laser Powder Bed Fusion (LPBF), and Electron Beam Melting (EBM) have gained prominence for their ability to fabricate high-performance metal components with intricate geometries and enhanced mechanical properties. These methods enable precise control over microstructural characteristics, allowing for improved heat transfer and wear resistance in tooling applications [42]. There have also been studies exploring the integration of different AM production techniques into a single production line, enabling greater flexibility and efficiency in manufacturing processes [43]. The integration of these methods into tool manufacturing has proven particularly valuable in addressing challenges associated with thermal management and material efficiency, further solidifying AM as a transformative solution in the manufacturing landscape. By utilizing these capabilities, AM not only addresses the thermal management challenges faced by conventional tooling methods but also opens new possibilities for innovation in mold design and manufacturing.

In the current study, a specialized sealing technique was developed for the RTM process, requiring different heating zones to achieve optimal performance. This challenge was addressed using two distinct mold designs. The first design utilized insulation materials to isolate heat and maintain the required thermal gradient, while the second design employed an innovative approach with inbuilt cooling channels directly integrated into one of the mold components. This component, produced using the Selective Laser Melting (SLM) process, featured a unique design with cooling channels on one side, acting as thermal insulation by controlling heat flow, and a lattice structure on the opposite side to retain and focus heat, enabling precise thermal management in critical areas. Positioned between these two functional layers was a heating element, which ensured precise thermal control in critical areas of the mold. In the current study, these two approaches were compared to evaluate their effectiveness in maintaining thermal conditions for the RTM process while preventing resin leakage. The functionality of both sealing configurations was validated by producing a hybrid metal–CFRP shaft and characterizing its mechanical properties. The integration of advanced cooling channels, lattice structures, and additive manufacturing highlights the potential for optimizing the RTM process with innovative thermal management and sealing solutions.

## 2. Materials and Methods

### 2.1. Self-Sealing Process Concept

In the traditional RTM process, polymer sealing is used along the mold contours to prevent matrix leakage. Matrix systems used in RTM are highly reactive, causing the polymer sealing to experience chemical stresses along with thermal stresses from production temperatures and mechanical stresses from mold closure forces. Over time, these combined stresses significantly reduce the sealing effectiveness, requiring regular replacement after a certain number of cycles. This increases production cycle times and leads to material wastage. As an alternative to traditional polymer sealing, a technique based on accelerated curing of the matrix, combined with a reduced mold cross-section and the possible use of catalysts to increase flow resistance, is proposed. This technique results in a self-sealing mechanism where the matrix cures near the mold contour, effectively sealing the cavity without the need for additional sealing materials. In vacuum-assisted processes, polymer sealing can still be used in areas exposed to lower thermal and mechanical stresses. This approach eliminates downtime associated with replacing sealing materials.

**Figure 3:**
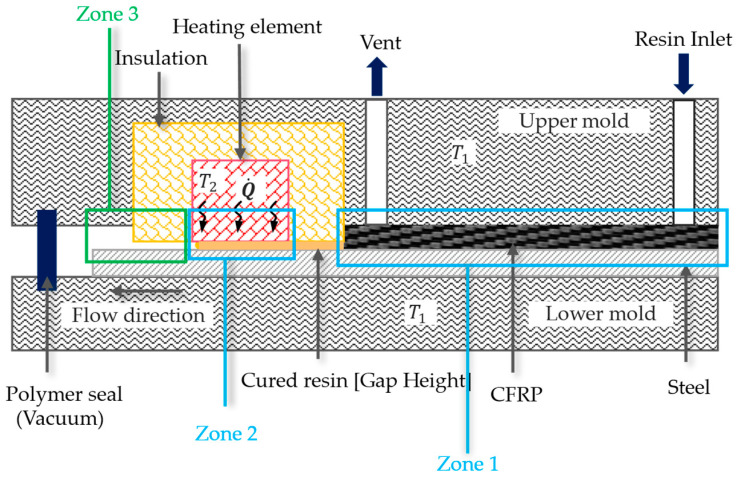
Block diagram showing the cross-section setup of self-sealing process in a VA-RTM Mold.

To validate the self-sealing concept, a mold was designed based on thermal and theoretical calculations. The mold was divided into two temperature zones. Zone 1 was designed to provide the temperature (T_1_) needed for manufacturing the sample, while Zone 2 provided a higher temperature (T_2_) for sealing at the mold contour, replacing traditional sealing materials in the VA-LRTM process. To prevent heat transfer from the self-sealing area (Zone 2) to the production area (Zone 1), a special insulation material was installed between the two zones. The self-sealing area included a rigid heating element and thermal insulation. The heating element was designed to deliver significantly higher temperatures at the mold contour compared to the rest of the mold, enabling the matrix to cure and seal effectively. The insulation material ensured that the main mold did not overheat, maintaining the required thermal gradient between the zones. Figure 3 shows the cross-section block diagram of the working principle of self-sealing in intrinsic manufacturing technique, replacing traditional polymer sealing in Zones 2 and 3. This concept is applicable to processes involving low-viscosity matrix injection (10–500 mPas).

### 2.2. Material Selection

Material selection in this study is divided into two sections: the materials for constructing the mold and those required for producing a hybrid component using the constructed mold. A precise approach was taken to ensure compatibility between the materials and the requirements of the RTM process, with specific attention to sealing methods, thermal zones, and the properties of the hybrid component.

### 2.3. Materials for Mold Construction

The mold in this study required two distinct heating zones that needed to be precisely controlled to facilitate the process. To achieve this, insulation materials were incorporated. Two types of insulation materials were selected: K-Therm^®^ AS600 and K-Therm^®^ AS55 (AGK-Hochleistungswerkstoffe GmbH, Dortmund, Germany). The K-Therm^®^ AS M series is known for providing electrical insulation at temperatures up to 800 °C. These materials are made from mica crystal structures, such as muscovite and phlogopite, and are pressed under high pressure and temperature using silicone resin as an adhesive. In this study, K-Therm^®^ AS was specifically chosen due to its low water absorption and ability to withstand temperatures up to 300 °C, making it ideal for validating the self-sealing process. This selection ensures stable insulation performance under high thermal loads. The insulation materials were precisely machined using a specialized ultrasonic cutting machine (DMG MORI Ultrasonic 65 MB, Bielefeld, Germany) due to their sensitivity to conventional milling processes, which can cause damage from vibrations. For the mold material, Tool Steel 1.2085 was used due to its corrosion resistance, an essential requirement in injection molding applications. Additionally, Tool Steel 1.2709 was utilized to fabricate the cooling channel component using the SLM process (DMG MORI LT30, Bielefeld, Germany), ensuring precise thermal management and compatibility with the mold’s operational requirements. This material offers thermal conductivity in the range of 15–20 W/m-K, aligning with the thermal management requirements of the mold and ensuring compatibility with the system (Table 1).

### 2.4. Materials for Hybrid Component Production

**Figure 4:**
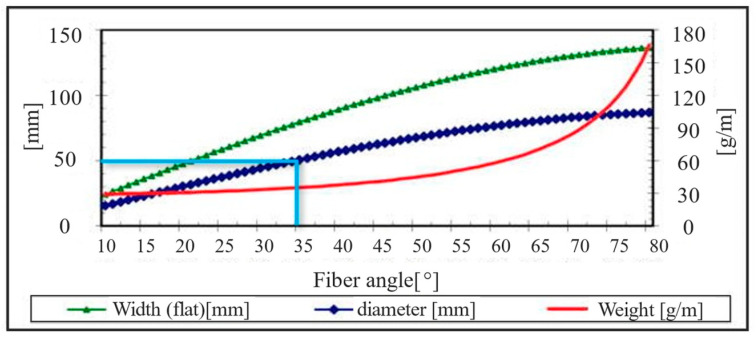
Angle of fibers with respect to different diameters.

The hybrid component produced in this study consisted of both metal and FRP materials. For the metallic portion, precision steel tube E235 + C with an outer diameter of 50 mm and a thickness of 1 mm was selected. The steel tubes were prepared using sandblasting (SMG 25 Duo, MHG Strahlanlagen GmbH, Düsseldorf, German), utilizing sand particles sized 210–297 μm at a pressure of 4–4.5 bars to increase surface adhesiveness. For the fiber-reinforced portion, Torayca^®^ T300J 400 tex (6k) carbon fiber dry fiber tubes were used. They have a fiber angle of 45° with a diameter of 60 mm and a thickness of 0.27 mm. The fiber angle could be adjusted by stretching or compressing the fiber tube, which consequently changed the diameter. In this study, the diameter varied between 50 and 54 mm, resulting in fiber angles between 35° and 37°, as shown in Figure 4. The figure is sourced directly from the data sheet provided by R&G Faserverbundwerkstoffe GmbH (Waldenbuch, Germany). The matrix system used for bonding was EPIKOTE Resin 05475 with the amine hardener EPIKURE Curing Agent 05443, both manufactured by Momentive Performance Materials Quartz GmbH (Geesthacht, Germany). This matrix system features low viscosity and a short curing time, which are critical for the RTM process. The viscosity of the resin-hardener mixture was temperature-dependent, as detailed in Table 2, with higher initial temperatures accelerating the curing process. The table is sourced directly from the data sheet provided by Lange + Ritter GmbH. A powdered version of EPIKOTE Resin 05475 was employed as a binder during the preforming stage of the dry fibers. The binder was applied in amounts corresponding to 1–2% of the total matrix system weight.

### 2.5. Sample Production

**Figure 5:**
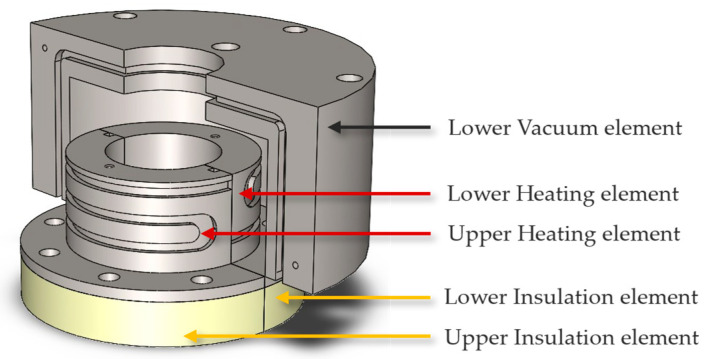
Design of mold insert for the self-sealing process, incorporating insulation material/Type 1.

**Figure 6:**
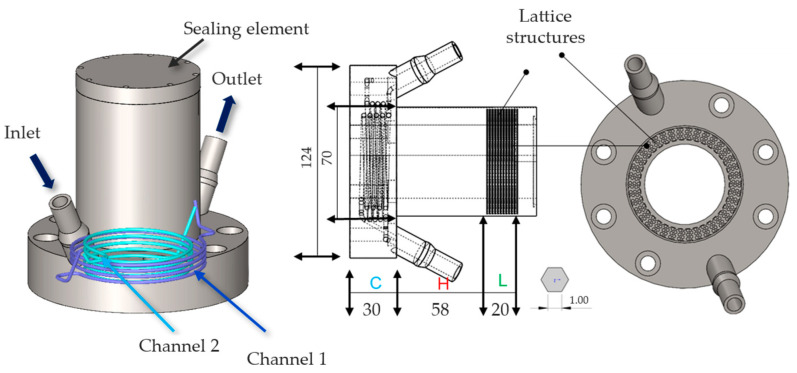
Design of mold insert for the self-sealing process utilizing cooling channels/Type 2 (C), a heating element (H) for localized high temperatures, and a lattice structure (L) for heat retention and distribution.

A mold was designed with insert features to allow for the validation of both sealing techniques. Instead of creating two separate molds, we designed a single mold with an insert feature, allowing the same mold to validate both types of self-sealing processes while retaining 95% of the original mold structure unchanged. Two types of inserts were developed to address the challenges of thermal management during the self-sealing process. Insert 1 was based on insulation materials and consisted of three key components: an insulation element made of K-Therm^®^ AS to prevent heat transfer from the self-sealing zone to the production area; a heating element to deliver high temperatures at the contour using deformable heating cartridges; and a vacuum element to create a vacuum and collect resin overspill in case of self-sealing failure. While effective in isolating heat and managing resin spills, Insert 1 required six components, which increased complexity, as shown in Figure 5. The components were split equally between the lower and upper molds for ease of production and installation into the mold. To simplify the design and improve thermal efficiency, Insert 2 was developed as an alternative. This design utilized a cooling channel on one side to block heat transfer from the heating zone to the mold zone, while a lattice structure on the opposite side retained heat and reduced energy dissipation. This optimized design not only addressed the limitations of Insert 1 but also enhanced energy efficiency and process reliability, as shown in Figure 6, with the number of components reduced to only two. Designing the cooling channel was a critical aspect of ensuring effective thermal management and maintaining the required temperature gradients in the mold. For this design, cooling channels with a fixed diameter of 3 mm were selected to eliminate the need for support structures during manufacturing, ensuring smooth surfaces and minimizing flow resistance. Two channels, each approximately 900 mm long, were designed to run in opposite directions, providing uniform cooling across the heated area and preventing the mold temperature from exceeding 120 °C. The length of the cooling channels was determined through theoretical calculations, which were based on Equation (1).

(1) L=Q˙4α∗Usemicircle∗∆T

**Figure 7:**
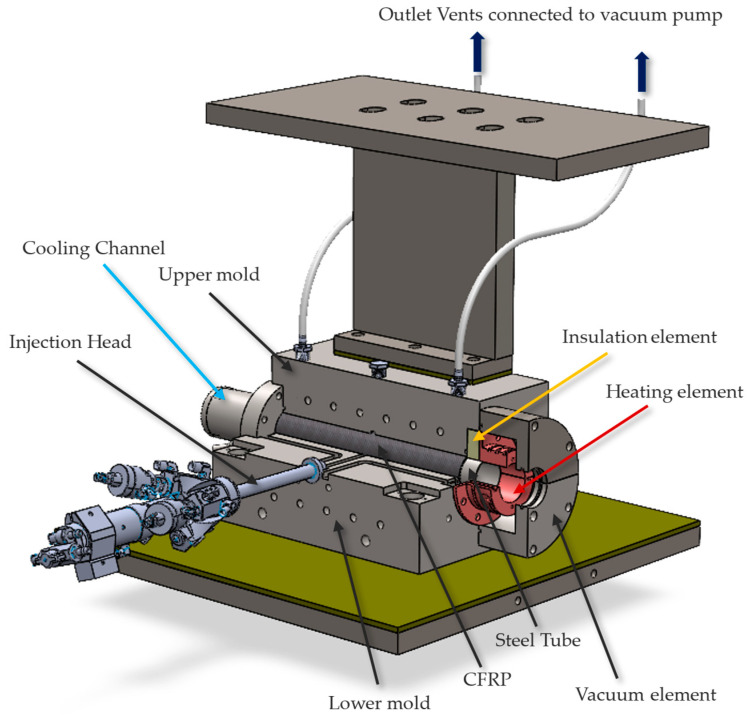
CAD model of the RTM mold, featuring a cooling channel insert on the left side for advanced thermal management and an insulation material insert on the right side to maintain thermal isolation.

In this equation, α represents the heat transfer coefficient; A is the area of heat transfer, and ΔT is the temperature difference between the wall and the fluid. The dimensions were carefully selected to ensure the system could dissipate the heat generated by the heating element at a water flow rate of 7.5 L/min. These calculations allowed for balancing cooling efficiency while maintaining the heating element temperature at 250 °C, which is critical for effective resin curing. Any necessary adjustments to the water flow rate can be made during experiments using a regulator valve to achieve precise thermal control. On the opposite side of the cooling channels, a lattice structure was incorporated to enhance thermal efficiency further. This structure reduces heat dissipation and redirects energy toward the resin curing area, ensuring optimal thermal management. The CAD model of the mold integrated with both inserts is shown in Figure 7, demonstrating its seamless integration into the existing VA-LRTM mold system.

## 3. Results and Discussion

### 3.1. Steady State Thermal Analysis

**Figure 8:**
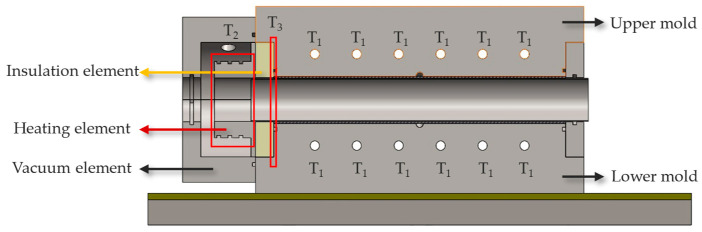
Cross-sectional view of the CAD model illustrating the boundary conditions applied during the FEM analysis.

**Figure 9:**
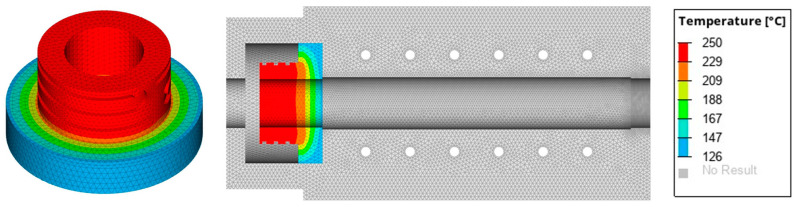
Steady-state analysis results of the mold with 20 mm thick AS 600 insulation material for the self-sealing process.

Effective thermal management is critical for ensuring the reliability and performance of the self-sealing process, necessitating a thorough steady-state thermal analysis to optimize the insulation material and its thickness. The thickness of the insulation material was determined based on steady-state thermal analysis performed using the Finite Element Method (FEM). In the current study, HyperWorks/OptiStruct was utilized to conduct the simulations. Material properties for the analysis were obtained from the respective manufacturers and are listed in Table 3. The simulation modeled both convection and conduction heat transfer to closely replicate real-world conditions. Convection was used to simulate heat transfer to the surrounding environment at a room temperature of 25 °C, while conduction represented heat flow between the various mold components, including heat transfer from the heating cartridge. The CAD model used for the simulation, along with the applied boundary conditions, is shown in Figure 8. The input parameters for the simulations included the production temperature (T_1_) and the self-sealing temperature (T_2_), while the contact temperature (T_3_) was determined through FEM analysis. Five simulations were performed with the same element size of 1 mm, varying the thickness and type of insulation material. Table 4 presents the contact surface temperatures of the mold for each simulation, where the goal was to maintain a temperature close to the production temperature, set at 120 °C. The results indicate that a 20 mm thickness of AS 600 insulation material maintained the contact temperature at approximately 126 °C, as shown in Figure 9. Based on these findings, the insulation thickness was increased to 25 mm for the self-sealing process to further enhance thermal performance. To validate the simulation results and ensure reliability, an experimental thermal analysis was conducted as the next step.

### 3.2. Transient State Thermal Analysis

**Figure 10:**
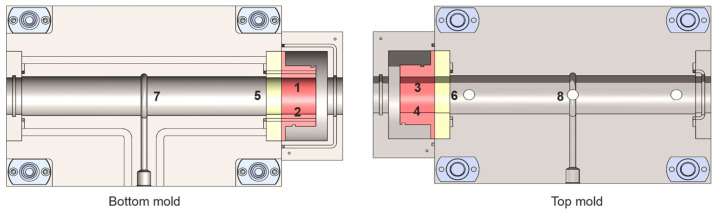
Experimental thermal analysis showing thermocouple locations in relation to the mold.

Before production, the mold was tested experimentally for transient-state thermal analysis to ensure the required temperature conditions were achieved for both types of inserts. The mold temperature was set to 120 °C, and the temperature in the heating element of the self-sealing zone was set to 250 °C for both insert types. To monitor the temperature over time, a total of eight thermocouples were used in addition to the thermocouple located in the heating element of the self-sealing and the mold. The extra thermocouples were soldered to a metal shaft, which was placed into the mold cavity to ensure direct contact with the mold surface. This method was chosen to avoid damaging the mold cavity and for simplicity. Thermocouples 1, 2, 3, and 4 were positioned at the center of the heating element in the self-sealing zone. Thermocouples 5 and 6 were placed 5 mm away from the insulation material toward the mold center, while thermocouples 7 and 8 were located 5 mm away from the center of the mold, as shown in Figure 10. For insulation material with a thickness of 25 mm, after 38 min, the temperature at the production zone center (locations 7 and 8) reached 120 °C. At the production zone ends (locations 5 and 6), slightly higher temperatures of 126 °C and 127 °C were recorded. The heating element in the sealing process successfully reached 250 °C, as shown in Table 5. Insulation with a thickness of 20 mm was also tested, and the recorded data are listed in Table 5. The test was extended for an additional 7 min, during which the temperature at all measured points remained homogeneous within a tolerance of ±3 °C.

**Figure 11:**
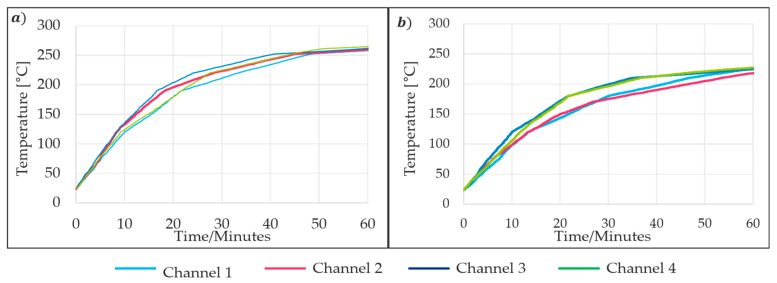
Experimental thermal profile of the mold with respect to water flow rate of (**a**) 2 L/min and (**b**) 3.5 L/min.

For the Type 2 insert, which uses cooling channels, the suitable water flow rate was determined experimentally using transient-state thermal analysis. A regulatory valve was used to control the water flow into the cooling channels to achieve the required temperature conditions that can adjust the flow rate from 1 to 8 L/min. The same thermocouple placement and methodology used for the Type 1 insert were applied to the Type 2 insert during the thermal trials. Both water flow and heating were activated simultaneously. At a water flow rate of 3.5 L/min, the heating zone did not reach 250 °C within 60 min, which is double the time required for the insulation-based self-sealing process, as shown in Figure 11. The flow rate was then reduced to 2 L/min, which is the minimum flow rate supported by the building’s water system. At this rate, the sealing zone reached 250 °C after 40 min, meeting the required condition. The outlet water temperature was measured at 18 °C, compared to an inlet temperature of 15 °C. However, these values may vary depending on environmental conditions and the operation of other water inlets within the central system.

### 3.3. Hybrid Sample Production

**Figure 12:**
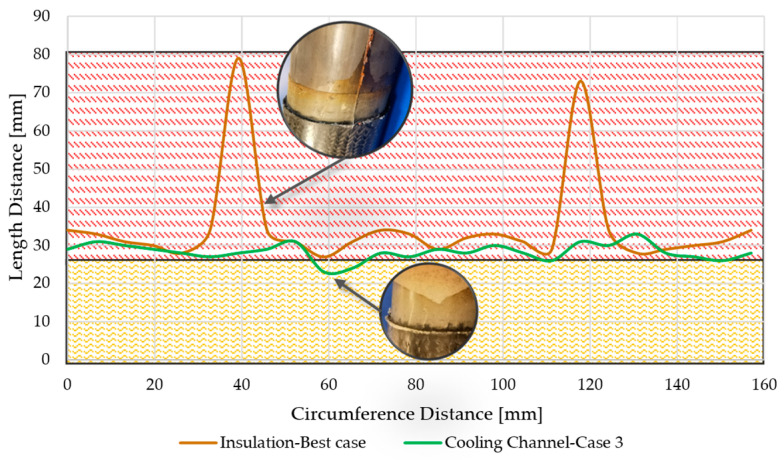
Resin profile in the self-sealing zone for insulation and cooling channel setups: Yellow indicates the insulation/cooling zone, and Red indicates the heating zone.

**Figure 13:**
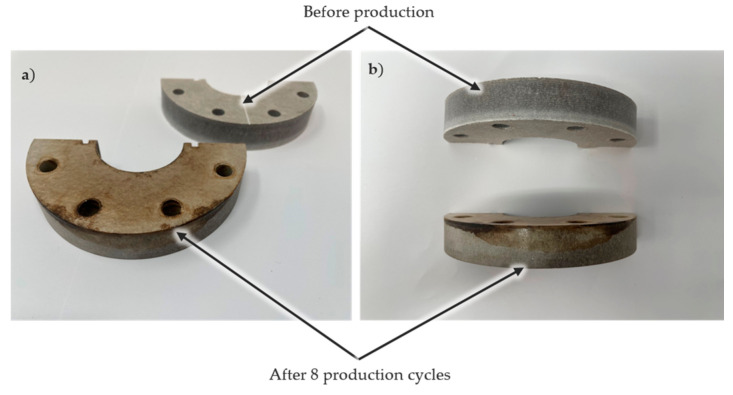
(**a**) Side view of insulation material contamination and (**b**) Bottom view of insulation material contamination.

Hybrid shafts were produced to validate the proposed self-sealing concept and to evaluate the efficiency of both types of inserts. The steel portion of the hybrid shaft sample was extended by 70 mm beyond the mold cavity on both ends. This extension allowed the shaft to stretch into the self-sealing zone, enabling the quantification of the self-sealing process’s efficiency and the capture of resin flow for comparison. The efficiency of the self-sealing process was assessed based on the area of resin deposited in and beyond the heating zone, with the height of the resin flow ignored for simplicity. A total of nine hybrid samples were produced using self-sealing via VA-LRTM with an insulation-based insert located on the right side and a cooling channel located on the left side of the mold. It should be noted that a standard silicone sealing was applied along the length of the mold to create a more controlled system. This ensured that the proposed self-sealing RTM process was utilized only at the mold ends. The insulation-based insert was placed on the right side of the mold, and the cooling channel insert was placed on the left side. A contour plot, shown in Figure 12, illustrates the resin flow length based on the circumferential position of the cylindrical shaft. The resin flow area was calculated by integrating these curves. Despite consistent production parameters, the sealing profiles of the nine samples varied significantly for the insulation-based insert. The calculated leakage area ranged from 1556 mm^2^ to 7527 mm^2^, corresponding to 15–71% of the total area available in the heating zone. For all nine samples, the primary leakage was consistently observed near the intersection of the upper and lower mold halves. This phenomenon can be attributed to variations in resin flow and curing energy requirements. At the intersection, the self-sealing zone is split into two sections, creating a secondary path for resin flow. Consequently, more resin accumulates in this area, requiring additional energy to cure effectively. This issue could potentially be resolved by replacing the split heating element in the self-sealing zone with a single-part heating element. Another major defect was observed in the insulation zone of the self-sealing process. Figure 13 shows the condition of the insulation material before and after eight production cycles. The side of the insulation material facing the mold was heavily contaminated due to epoxy infiltration into the porous glass fleece, which significantly affected its thermal performance. Approximately 25% of the insulation material was infiltrated with epoxy, reaching areas not intended to encounter resin. In contrast, for the AM prototype insert, the resin flowed to a maximum distance of only 6 mm under the heating zone, making it a better option compared to the insulation-based self-sealing process. Additionally, no contamination of the cooling channel was observed, even after nine production cycles, highlighting its superior durability and performance.

**Figure 14:**
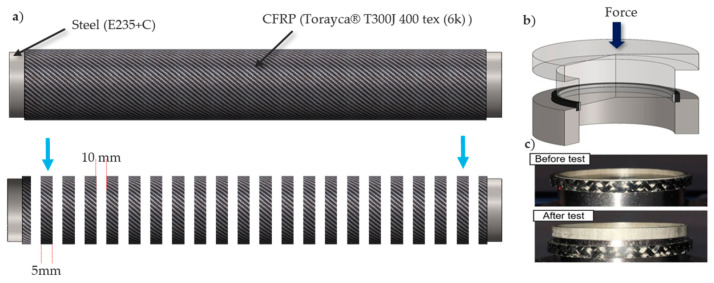
(**a**) Pictorial representation of the specimens with respect to the sample; (**b**) Test rig used to perform ILSS testing; and (**c**) Specimens before and after testing.

**Figure 15:**
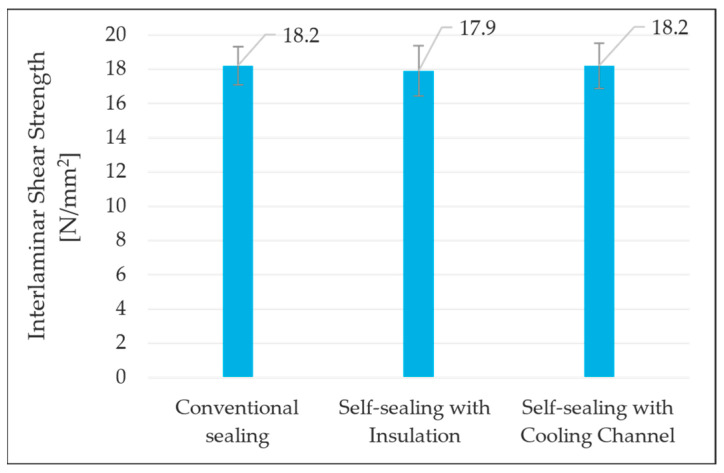
ILSS results of hybrid shafts manufactured using different sealing setups.

To evaluate whether the self-sealing process influenced the mechanical properties, Interlaminar Shear Strength (ILSS) testing was conducted. A specialized ILSS testing method was developed for cylindrical shafts, adapted from standard shear strength testing of flat composite specimens. Each hybrid shaft was cut into 20 rings, each with a thickness of 5 mm. These rings were inserted into a shear-edge device, which pressed the steel ring out of the CFRP structure. The recorded force was normalized to the shear surface (steel/CFRP interface) to determine shear strength. By designing the die and punch appropriately, the force flow was directed precisely through the interface to ensure a well-defined stress state. Figure 14 illustrates the ILSS test setup and specimens before and after testing. The results are based on the average values of one specimen per sample across five samples, with standard deviations represented as error bars. Specimens near the sealing zone, indicated by a blue arrow in Figure 14, were analyzed in detail, and ILSS values are shown in Figure 15. The results indicate that the AM prototype did not have any significant negative impact on the ILSS values compared to the insulation-based self-sealing and traditional sealing setups. This confirms that the AM prototype is a viable replacement for existing sealing materials, providing a cost-effective solution while advancing toward an optimized manufacturing process.

## 4. Conclusions

This study successfully validated the proposed self-sealing concept for the VA-LRTM process by producing hybrid shafts using two distinct insert types: an insulation-based insert and an AM-manufactured cooling channel insert. The insulation-based insert demonstrated variability in sealing profiles, with leakage areas ranging from 15% to 71% of the heating zone’s total area. Additionally, the porous insulation material faced significant contamination from epoxy infiltration, affecting its thermal performance and requiring frequent replacements after repeated production cycles. Post-production cooling for the insulation-based insert took approximately 4 h, further highlighting its limitations in process efficiency. In contrast, the AM cooling channel insert showcased significant advantages. Resin flow under the heating zone was limited to 6 mm, and no contamination was observed even after nine production cycles. Notably, the AM cooling channel allowed the mold to cool to room temperature within 20 min, drastically reducing production downtime compared to the insulation-based insert. These findings demonstrate the AM insert’s superior durability, thermal efficiency, and process reliability, making it a sustainable and scalable solution.

Mechanical testing through ILSS analysis further confirmed that the AM prototype had no significant negative impact on the mechanical properties of hybrid shafts when compared to the insulation-based self-sealing and traditional sealing setups. The AM-manufactured insert not only improved thermal management and contamination resistance but also demonstrated the potential to streamline production cycles and reduce operational costs. Its design flexibility, durability, and precision in maintaining targeted thermal gradients make it an ideal candidate for replacing conventional sealing materials. Furthermore, the integration of AM into the VA-LRTM process represents a significant step toward automation and enhanced efficiency in composite manufacturing.

This study highlights how additive manufacturing can optimize injection molding tool design, focusing on improved thermal performance and enhanced process efficiency, thereby providing a reliable and effective solution for advanced composite manufacturing.

## Figures and Tables

**Table 1:** Mechanical properties of the tool steel—1.2709.

Tensile Strength [MPa]	Yield Strength [MPa]	Elongation at Break [%]	Hardness [HV10]	Surface Roughness [R_a_]
1095	945	11	550	5

**Table 2:** Material properties of epoxy and steel shaft.

**EP 05475 + EK 05443 (100:24)**		
Viscosity [mPa.s]	at 25 °C	1200 ± 100
	at 80 °C	30 ± 5
	at 100 °C	13 ± 3
Pot Life [min]	at 25 °C	120 ± 10
Gel Time [s]	at 80 °C	330 ± 30
	at 100 °C	210 ± 30
	at 120 °C	150 ± 30
	at 140 °C	90 ± 30
**E235 + C**		
Yield strength [MPa]	235–355	
Tensile strength [MPa]	480–640	
Elongation at break [%]	≥4–6	

**Table 3:** Material parameters used for steady-state analysis.

Materials	Young’s Modulus [E]	Poisson’s Ratio [υ]	Density [ρ]	Thermal Conductivity [k]	Specific Heat Capacity [H]
	GPa	-	kg/(m^3^)	W/(m × K)	J/(kg × K)
Tool Steel—1.2085	210	0.3	7.85 × 10^3^	20	508
AS 600	6	0.4	2.2 × 10^3^	0.25	1200
AS 550	4	0.4	1.8 × 10^3^	0.37	1280
CFRP (CF + Epoxy)	129	0.32	1.55 × 10^3^	3.9	900
Resin	2.35	0.4	1.2 × 10^3^	0.18	1180
Silicon Sealing	0.05	0.49	1.44 × 10^3^	0.3	1100

**Table 4:** FEM results showing contact surface temperatures of the mold for different insulation materials and thicknesses.

Insulation Material	Thickness [mm]	Temperature, T_3_ [°C]
AS 600	12	142
	15	131
	20	126
AS 550	15	153
	20	146

**Table 5:** Experimental data recorded at 40 min across 8 locations for both inserts, with two different parameters for each type (note: values are averaged from 5 data points per location).

Insert Type	Channel 1 [°C]	Channel 2 [°C]	Channel 3 [°C]	Channel 4 [°C]	Channel 5 [°C]	Channel 6 [°C]	Channel 7 [°C]	Channel 8 [°C]
Type 1 @ 20 mm	252	251	251	253	134	139	122	121
Type 1 @ 25 mm	251	253	251	251	126	127	118	121
Type 2 @ 3.5 L/min	213	217	215	217	97	98	119	118
Type 2 @ 2 L/min	247	251	249	251	121	123	119	121

## Data Availability

The original contributions presented in this study are included in the article. Further inquiries can be directed to the corresponding author.
